# The improvement of healthy habits in patients with severe mental disorders: the LIFESTYLE trial

**DOI:** 10.1192/j.eurpsy.2023.243

**Published:** 2023-07-19

**Authors:** V. Sollo, M. Di Vincenzo, M. Carbone, A. Rosa, R. Toricco, L. Tretola, M. Luciano, G. Sampogna, A. Fiorillo

**Affiliations:** Psichiatry, Università della Campania Luigi vanvitelli, Naples, Italy

## Abstract

**Introduction:**

The impact of unhealthy lifestyle behaviors is significant in the general population, being associated with chronic physical conditions, reduced life expectancy and increased healthcare costs. This impact is higher in patients with severe mental disorders (SMD). In fact, SMD patients present higher rates of obesity, metabolic syndrome, diabetes, and cardiovascular diseases compared to the general population. The relationship between physical and mental health is multifactorial and includes side effects of many psychotropic drugs, sedentary behaviors, reduction of physical exercise, smoking, and substance abuse. Finally, illness-related factors, including cognitive impairment, reduced psychosocial functioning, social isolation, and self-stigma, can significantly impact on patients’ physical health.

**Objectives:**

This study, coordinated by the Department of Psychiatry of the University of Campania “L. Vanvitelli”, aims to test the efficacy of a lifestyle group intervention, compared to a brief psycho-educational intervention, in improving healthy habits in a real-world sample of patients with SMD.

**Methods:**

401 patients were recruited and randomly allocated to receive the experimental or the control intervention. Inclusion criteria were age between 18 and 65 years; primary diagnosis of schizophrenia, schizoaffective disorder, delusional disorder, other psychotic disorders, major depressive disorder, or bipolar disorder according to the DSM-5; BMI≥ 25. At baseline and 6 months post-randomization all patients were administered: SCID-5, BPRS, MATRICS, MCCB, IPAQ and a questionnaire on lifestyle behaviors developed by the Italian National Institute of Health.

**Results:**

206 patients were allocated to the experimental group and 195 in the control one, of which 43.3% had a main diagnosis of bipolar disorder, 29.9% of psychosis and 26.9% of major depression. Patients were mainly female (57%), with a mean age of 45.6±11.8 years and with an educational level of 11.7±2.9 years. All patients were treated with at least one psychotropic drug. About 29.4% of patients reported performing physical activity regularly, while only 3.7% performed at least 75 min of vigorous physical activity per week. Patients practicing physical activity report higher levels of perceived satisfaction with the quality of life compared with non-active patients (p < 0.005). A general improvement in dietary patterns from T0 to T1 was found in patients receiving the experimental intervention. We found an increased weekly intake of fish (*p* < .001), vegetables (*p* < .05) and fresh fruit (*p* < .01). Moreover, we also found a reduction of junk food (*p* <.05) and of weekly consumption of cereals (*p* < .01).

**Conclusions:**

Our findings show that patients with SMD can improve their lifestyle behaviors with appropriate support. There is the need to implement similar interventions clinical practice to reduce the mortality gap in patients with SMD.

**Disclosure of Interest:**

None Declared

